# Microbiota and Cheese Quality

**DOI:** 10.3390/foods15162796

**Published:** 2026-08-10

**Authors:** Juan A. Centeno, Javier Carballo

**Affiliations:** 1 Food Technology Area, Faculty of Sciences, University of Vigo, 32004 Ourense, Spain; carbatec@uvigo.es; 2 Instituto de Agroecoloxía e Alimentación (IAA), University of Vigo, Campus Auga, 32004 Ourense, Spain

## 1. Introduction

Few foods exemplify the beneficial role of microorganisms as comprehensively as cheese. Indeed, microorganisms are the primary drivers of cheese diversification, contributing not only to its preservation, nutritional value, and functional properties, but also to the development of the sensory characteristics that distinguish the extraordinary diversity of cheese varieties. It has been estimated that between 1000 and 1500 cheese varieties exist worldwide [1]. With the exception of sweet (non-acidified) fresh cheeses produced exclusively through rennet-induced coagulation of pasteurized milk, such as the Spanish Burgos cheese, and pasteurized fresh white cheeses such as the Latin American-style *Queso Blanco*, virtually all cheese varieties—whether acid-coagulated, enzyme-coagulated, or produced through a combination of both mechanisms—depend on the deliberate addition of microorganisms or on the activity of the indigenous microbiota naturally present in raw milk. These microbial communities are responsible not only for acidification and fermentation, but also for the ripening processes associated with the metabolism of milk constituents, including lactose, citrate, proteins, and lipids, ultimately giving rise to the sensory properties that define each cheese variety.

Cheese quality can be evaluated in terms of three complementary dimensions: microbiological quality and safety, nutritional quality, and sensory quality. Although these attributes are often considered independently, they are all intrinsically linked to the cheese microbiota, a dynamic and highly adaptive ecosystem composed of intentionally added microorganisms together with the indigenous and adventitious microbiota naturally associated with milk and the cheesemaking environment [2,3]. From this perspective, the cheese microbiota should not merely be regarded as a technological tool, but rather as the biological foundation upon which cheese quality is built. Through complex microbial interactions and metabolic activities, this ecosystem ultimately determines product safety, influences nutritional and functional value, and drives the biochemical transformations responsible for the development of the distinctive sensory profile of each cheese variety. Accordingly, understanding how the cheese microbiota shapes these three interconnected dimensions of quality has become a cornerstone for the development of the next generation of safer, healthier, and higher-quality cheeses.

The microbiological quality of cheese, particularly with regard to food safety, is primarily determined by the absence of foodborne pathogens and toxic metabolites of microbial origin. Nevertheless, cheese constitutes a complex microbial ecosystem in which safety depends not only on the exclusion of undesirable microorganisms, but also on the establishment of a balanced microbiota capable of limiting their growth. The principal microbial hazards associated with cheese include foodborne pathogens such as *Listeria monocytogenes*, *Salmonella* spp., enteropathogenic *Escherichia coli*, and *Staphylococcus aureus* [4]. In addition, the growth of contaminating microorganisms may lead to the accumulation of mycotoxins and biogenic amines, both of which represent significant concerns for consumer health. Importantly, it should not be overlooked that fermented foods, particularly those produced through spontaneous fermentation, may also serve as reservoirs of antimicrobial resistance genes, an emerging food safety issue that deserves increasing attention [5].

Within this context, the cheese microbiota plays a dual role. On the one hand, lactic acid bacteria (LAB) act as natural biopreservatives, enhancing food safety while reducing the need for synthetic preservatives, thereby contributing to more sustainable food systems [6]. Numerous LAB strains also produce bacteriocins with potent antagonistic activity against major foodborne pathogens [7]. On the other hand, certain intentionally added or adventitious LAB and yeast strains exhibit amino acid decarboxylase activity, leading to the formation of biogenic amines such as histamine and tyramine. Therefore, the careful selection of starter and adjunct cultures, together with appropriate control of ripening conditions, is essential to minimize biogenic amine accumulation in dairy products [8].

Mold growth on the surface of cheese varieties that are not intentionally mold-ripened is generally considered undesirable because of the potential production of mycotoxins [9]. Conversely, selected yeasts can be used as bioprotective cultures to inhibit the growth of spoilage and toxigenic fungi in fermented foods, thereby contributing to product safety [10].

The traditional concept of cheese’s nutritional quality has progressively evolved toward one of functional quality, reflecting the growing recognition that cheese can provide health benefits beyond its nutritional value. This functional dimension is largely determined by the cheese microbiota, which not only enables cheese to serve as an effective carrier of probiotic microorganisms, but also generates a wide range of bioactive compounds with antioxidant, antihypertensive, immunomodulatory, antimicrobial, anti-adipogenic, and cardioprotective properties.

Cheese is widely regarded as one of the most suitable food matrices for the delivery of probiotic microorganisms because of its physicochemical characteristics and its ability to maintain microbial viability during storage and gastrointestinal transit [11,12]. Accordingly, probiotic bacteria, such as *Lactobacillus acidophilus* and *Bifidobacterium* spp., have been successfully incorporated as adjunct cultures in fresh and short-ripened cheeses to enhance their functional properties [13]. Likewise, *Saccharomyces cerevisiae* var. *boulardii* has attracted increasing interest because of its ability to inhibit intestinal pathogens and promote gut health [14].

Among the most relevant bioactive compounds produced by *Lactococcus lactis* and other LAB are exopolysaccharides (EPSs), bioactive peptides, conjugated linoleic acid, γ-aminobutyric acid (GABA), vitamins, and enzymes [8]. In addition, selected probiotic LAB have been shown to significantly reduce the accumulation of biogenic amines, further enhancing the functional and microbiological quality of cheese [15].

Finally, sensory quality encompasses the organoleptic attributes of flavor, texture, and appearance, as well as the absence and control of sensory defects and off-flavors. Among the three dimensions of cheese quality, sensory quality is arguably the one most directly shaped by the cheese microbiota. Through a wide array of metabolic and enzymatic activities—including pigment production, carbohydrate and citrate fermentation, proteolysis, lipolysis, and the subsequent catabolism of amino acids and free fatty acids—microbial communities drive the biochemical processes that ultimately define the sensory identity of each cheese variety.

The distinctive sensory signature of a particular cheese is shaped from the very beginning by the microbial quality and diversity of the milk from which it is produced. In modern dairy production, one of the major microbiological challenges is the proliferation of psychrotrophic bacteria, particularly in cow’s milk, as these microorganisms may grow during refrigerated storage and eventually dominate the bulk-tank milk microbiota [16]. Consequently, thermal treatments such as thermization or pasteurization are widely applied to reduce the psychrotrophic microbial load and limit spoilage [17]. In contrast, many artisanal raw-milk cheeses are traditionally manufactured without starter or adjunct cultures, relying entirely on spontaneous fermentation driven by the indigenous and adventitious microbiota. These cheeses are widely recognized for their richer and more distinctive flavor compared with cheeses produced from pasteurized or microfiltered milk, a characteristic largely attributed to the native microbiota of raw milk [18]. Likewise, the use of selected autochthonous microbial cultures has proven to be an effective strategy for reinforcing the authentic sensory characteristics of Protected Designation of Origin (PDO) cheeses while preserving their typicity and bringing them closer to their traditional counterparts [5,19,20].

Flavor development is orchestrated by the succession and metabolic interactions of distinct microbial populations throughout cheesemaking and ripening. The coordinated activities of starter lactic acid bacteria (SLAB), non-starter lactic acid bacteria (NSLAB), and the diverse secondary microbiota—including propionibacteria, *Micrococcaceae*, *Staphylococcaceae*, coryneform bacteria, yeasts (*Debaryomyces hansenii*, *Kluyveromyces* spp., *Yarrowia lipolytica*, and *Geotrichum candidum*), and molds—vary according to cheese variety and collectively govern the formation of the volatile and non-volatile compounds responsible for the characteristic flavor of each cheese [21]. Ultimately, cheese flavor is the metabolic fingerprint of its microbiota.

Beyond flavor development, the cheese microbiota also plays a decisive role in texture formation. Many SLAB produce CO_2_ through lactose and citrate metabolism, thereby contributing to the characteristic open texture of Dutch-type and related cheeses, whereas EPS-producing LAB enhance the viscosity and creaminess of fresh and short-ripened cheeses [22]. In smear-ripened and bloomy-rind cheeses, molds and certain yeasts metabolize lactic acid, increasing the surface pH to above 6.0. This rise in pH reactivates proteolytic enzymes, promoting progressive softening from the rind toward the center, a hallmark of these cheese varieties [23].

Recent advances in multiomics are transforming our understanding of cheese fermentation and ripening. Integrated genomic, transcriptomic, proteomic, metabolomic, and volatilomic approaches now enable comprehensive characterization of cheese microbial ecosystems and their metabolic functions. In particular, the autochthonous microbiota of artisanal cheeses can be characterized and preserved as microbial “DNA barcodes”, providing a valuable resource for linking microbial composition with sensory quality, safety, and geographical authenticity [22]. Likewise, the integration of genomics and metabolomics has enabled the association of individual microorganisms with specific volatile compounds, facilitating the identification of molecular biomarkers that explain the relationships between microbial communities, flavor development, and cheese quality [24,25]. Ultimately, these approaches will enable the rational selection of next-generation starter and adjunct cultures capable of simultaneously enhancing cheese safety, nutritional value, and sensory quality while preserving the typicity and authenticity of traditional cheese varieties [25].

## 2. Overview of the Published Articles

The articles included in this Special Issue provide complementary insights into key aspects of cheese microbiology and their impact on cheese quality, safety, technological performance, and sensory properties.

Despite the fact that microorganisms play a central role in determining the sensory characteristics and safety of cheese, some traditional cheese varieties remain under-studied in terms of their microbial profiles. This limits the possibilities for intervention to improve their quality. This was the case with hurood, a traditional unripened cheese prepared from raw milk using distinctive Mongolian (Inner Mongolia, China) artisanal techniques (milk coagulation without rennet addition and heat treatment of curd both before and after whey removal). To understand the microbial events taking place in this variety and to cover the diversity within it, Chen et al. [26] examined the microbial indicators, the community composition, and the succession dynamics across four manufacturing stages (raw milk, acid-coagulated curd or “yogurt”, whey, and hurood) across three production scales (pastoral household, workshop, and factory). Twenty-four samples (two from each sampling time in each manufacture type) were collected and analyzed to determine their physicochemical parameters (pH and Aw), the counts of some microbial groups (total aerobic bacteria, molds and yeasts, and coliforms) and bacterial diversity using total DNA extraction and 16S rRNA gene amplicon sequencing. Total counts and coliforms decreased as the manufacturing process unfolded, consistent with the combined effects of acidification and heat treatment. Regarding the effect of production scale, no significant effect on pH and Aw values was observed, suggesting that scale-dependent differences in microbiological quality may be associated primarily with hygiene management practices. Factory production exhibited a more favorable microbiological profile in the final product (lowest mold and coliform counts), whereas workshop-scale production showed the greatest enrichment of environmental contamination indicator bacteria. Proteobacteria was found to be the main phylum in raw milk, whereas Firmicutes dominated in curd, whey and cheese, with *Lactococcus* and *Lactobacillus* representing the dominant genera at these three sampling points. In addition, whey showed a high abundance of *Acetobacter*. This study provides valuable information for identifying critical control points and microbiological risks among each manufacturing type and establishes a basis for standardized production strategies that preserve the product’s uniqueness while improving safety and the consistency of its sensory attributes.

While the biochemical and microbiological characteristics of some traditional cheeses remain unknown and have only recently begun to be investigated, other cheese varieties have been thoroughly studied over recent decades. The microbiological and biochemical phenomena occurring during ripening are comparatively wellcharacterized in these cheeses, which are protected by a PDO and have already achieved both local and international prestige. For example, the characteristics of Cabrales cheese, a traditional Spanish blue cheese, have been extensively studied. Nevertheless, even in these well-characterized cheeses, an in-depth investigation of varieties exhibiting exceptional quality could help identify the microorganisms associated with the distinctive properties and sensory excellence sought by cheesemakers. To this end, Rodríguez et al. [27] carried out an in-depth microbiological and biochemical study of sixteen long-ripened, high-quality Cabrales cheeses from independent producers. Cheeses were analyzed to obtain counts of their main microbial groups (total aerobic mesophilic bacteria, lactococci, lactobacilli, enterococci, enterobacteria, coliforms, micrococci and staphylococci, and molds and yeasts), and to determine their organic acid, sugar, amino acid, biogenic amine, and volatile compound contents. In addition, a metataxonomic analysis was carried out by isolating the total microbial DNA of cheeses and subsequently amplifying and sequencing the ribosomal sequences. The authors identified positive and negative correlations between bacterial and fungal species, as well as between microbial populations and species and specific key biochemical markers. Based on these associations, the authors proposed a candidate starter mixture of bacterial (*Lactococcus lactis*, *Tetragenococcus koreensis*, *Staphylococcus equorum*, *Brevibacterium linens*, and *Corynebacterium variabile*) and fungal (*Penicillium roqueforti*, *Debaryomyces hansenii*, *Geotrichum candidum*, and *Kluyveromyces lactis*) strains for experimental testing; its capacity to improve Cabrales cheese quality remains to be demonstrated.

The search for, characterization of, and evaluation of novel microbial strains with outstanding metabolic capabilities for use as starter or adjunct cultures remain a priority for both the cheese industry and the scientific community. The discovery and use of new microbial strains allow for increased organoleptic diversity in cheeses while reinforcing the personality and uniqueness of popular traditional varieties. From this perspective, Jara et al. [28] evaluated the *Ligilactobacillus salivarius* SP36 strain (a strain isolated from a cheese seal which was last used in 1936 in a small dairy in the Spanish Pyrenees) as an adjunct or starter culture in ripened cheese manufacturing. For this purpose, three different cheese batches were manufactured: (i) C1 (control), in which milk was inoculated with a commercial starter culture containing *Lactococcus lactis* subsp. *lactis*, *Lc. lactis* subsp. *cremoris* and *Lc. lactis* subsp. *lactis* biovar. *diacetylactis*; (ii) C2, made with the same commercial starter plus *Lig. salivarius* SP36; and (iii) C3, made with only *Lig. salivarius* SP36. Cheeses from these three batches were ripened for 150 days and analyzed to determine their physicochemical parameters (pH, Aw, and color), volatile compounds, and microbiological safety (enumeration of *Salmonella* spp., *Listeria monocytogenes* and coagulase-positive staphylococci). In addition, metataxonomic analysis of the cheeses was carried out to study their microbial diversity, while isolates of LAB were obtained, identified by sequencing the 16S rRNA gene, and their safety characteristics (antibiotic resistance and biogenic amine production) were assessed. The authors observed that LAB dominated the three cheese types, with the cheese manufactured without the commercial starter showing the highest microbial diversity. *Ligilactobacillus salivarius* SP36 influenced the aroma profile of the cheese, increasing the ethyl esters, alcohols, ketones, organic acids and sulfur compounds. The authors concluded that *Lig. salivarius* SP36 is a promising adjunct culture for ripened cheeses, while some strains of *Lactiplantibacillus plantarum* and *Lacticaseibacillus paracasei* isolated from the cheeses could represent candidates for starter or adjunct cultures.

Cheese not only harbors microorganisms responsible for biochemical changes (both positive and negative) that are reflected in the organoleptic characteristics of the final product, but it also, in many cases, carries microorganisms that can directly or indirectly compromise consumer health. In recent years, the emergence of antibiotic-resistant bacteria and their transmission through food has become a major public health problem. These antibiotic-resistant bacteria can cause direct problems, but above all, they serve as reservoirs of resistance genes that can be transferred horizontally to other bacteria, severely compromising public health. Due to the process of their production—with the possibility of contamination from multiple sources—and their compositional characteristics, dairy products, particularly cheese, may act as vehicles for the transmission and dissemination of bacteria carrying antibiotic resistance genes. Studies on antibiotic-resistant bacteria in cheese are scarce and mainly focus on fresh cheeses. Therefore, the work by Arraiz-Fernández et al. [29] constitutes a valuable contribution to the scientific literature. The authors evaluated the microbiological quality of Spanish aged cheeses manufactured both from raw and pasteurized milk, and the antimicrobial resistance of enterococci, staphylococci, and *Enterobacterales* present in them. They collected 60 aged cheeses at the retail level produced in the north of Spain and analyzed them to obtain counts of mesophiles, LAB, staphylococci, enterococci, *Enterobacterales*, and yeasts using appropriate culture media. Then, three to six colonies were randomly selected from each duplicate sample and culture medium and identified at the species level using MALDI-TOF MS. Finally, the isolated strains of *Enterococcus* spp., *Staphylococcus* spp., *Mammaliicoccus* spp., and *Enterobacterales* were analyzed to determine their phenotypic resistance to panels of 16–35 antibiotics. As expected, LAB were the dominant group, with *Lactiplantibacillus lactis* representing the main species. Antimicrobial resistance was outstanding in *Enterococcus faecalis* strains isolated from raw-milk cheeses, of which 9.30% were multidrug-resistant. Multidrug-resistant *Staphylococcus equorum* strains were also isolated from both raw-milk (1.69% of the isolates) and pasteurized-milk (9.09%) cheeses. Among *Enterobacterales*, 37.5% of the isolates were multidrug-resistant. This study shows that such resistant enterococci, staphylococci, and *Enterobacterales* can be present in aged cheeses and highlights the need for strategies to control their presence throughout the cheesemaking process.

While most bacterial groups present in cheese have relatively well-characterized roles, certain genera have a diverse roles with highly variable effects, ranging from technologically beneficial to detrimental, as is the case with the genus *Staphylococcus*. Coagulase-positive staphylococci, primarily *S. aureus*, pose a danger to consumers due to the ability of some strains to produce enterotoxins that cause a well-known type of food poisoning. Some strains also carry antibiotic resistance genes and present a risk to public health. However, coagulase-negative staphylococci, a group that includes species such as *S. carnosus*, *S. condimenti*, *S. equorum*, *S. piscifermentans*, *S. succinus*, and *S. xylosus*, exhibit primarily proteolytic and lipolytic metabolic activities that play a significant role in the development of desirable sensory attributes in ripened cheeses. This dual role of *Staphylococcus* species in cheese is reviewed by Casagrande Ribeiro et al. [30]. The authors begin with a brief introduction to the history and evolution of knowledge regarding the role of staphylococci in food, and then proceed to describe the two clearly differentiated groups of staphylococci (coagulase-positive and coagulase-negative), along with their species and most relevant genetic and metabolic characteristics. After discussing the virulence factors of this genus, including biofilm formation, antibiotic resistance, and enterotoxin production, the work focuses in depth on staphylococcal food poisoning (SFP) associated with cheese consumption. It addresses the factors that affect the growth and enterotoxin production of *Staphylococcus* in cheese and describes cheese-consumption-related outbreaks of SFP reported in the literature. The manuscript concludes by discussing strategies for controlling staphylococci in cheese and the current microbiological criteria implemented around the world for *Staphylococcus* and enterotoxins in cheese, before drawing conclusions and highlighting some open questions regarding the role of the *Staphylococcus* genus in cheese.

Taken together, the articles gathered in this Special Issue confirm that the cheese microbiota is far more than a driver of fermentation. It is a key determinant of food safety, technological performance, and, ultimately, the sensory identity and typicity that distinguish each cheese variety. Continued advances in microbial ecology, omics technologies, and culture selection will undoubtedly contribute to preserving traditional cheeses while fostering innovation and sustainability in the dairy industry.

## Data Availability

No new data were created or analyzed in this study. Data sharing is not applicable to this article.
